# Preparation of Platycodin D Microspheres and Their Protective Effects on Type 2 Diabetes Mellitus

**DOI:** 10.3390/molecules31132305

**Published:** 2026-07-01

**Authors:** Jingjing Huang, Xiong Han, Lixia Yang, Qiong Shen, Yuxin Pang, Yanfei Li

**Affiliations:** 1 Pharmacy College, Guizhou University of Traditional Chinese Medicine, Guiyang 550025, China; 18885223220@163.com (J.H.);; 2 Blumea Balsamifera Research Center, Guizhou University of Traditional Chinese Medicine, Guiyang 550025, China; 3 Guizhou Research Center of Conservation and Utilization of Wild Resources of Karst Traditional Chinese Medicine and Ethnic Medicine, Anshun 561000, China; 4 State Key Laboratory of Subtropical Silviculture, College of Food and Health, Zhejiang A&F University, Hangzhou 311300, China

**Keywords:** type 2 diabetes mellitus, Platycodin D, natural compounds, antioxidant, pH-responsive

## Abstract

The treatment of type 2 diabetes (T2DM) faces numerous challenges. Oral insulin (Ins) and other short-acting compounds still encounter significant obstacles in the hostile gastrointestinal environment, including low bioavailability and rapid metabolic clearance. Platycodin D (PD) is a natural compound with demonstrated hypoglycemic and lipid-lowering effects. In this study, PD was encapsulated using alginate to prepare orally administrable, pH-responsive, gut-targeted gel microspheres (PD@MPs), and their efficacy in improving T2DM prognosis was investigated. In vitro release studies demonstrated that PD@MPs avoided degradation by gastric acid and were released in the intestine. Cell experiments indicated that PD possessed significant antioxidant and anti-apoptotic properties. Masson, immunohistochemistry, and immunofluorescence staining revealed that PD@MPs alleviated inflammation in key metabolic organs and maintained normal pancreatic tissue function and morphology. Western blot analysis assessed the expression of proteins related to hepatic glycogen synthesis, including IRS-1, GLUT2, GSK-3β, and AKT. The research results indicate that the assembly strategy using sodium alginate (SA) as the coating layer has enabled the oral administration of PD and has demonstrated its potential in the treatment of diabetes.

## 1. Introduction

Diabetes mellitus (DM) is a chronic metabolic disorder characterized by impaired glucose and lipid metabolism, encompassing various types, including type 1 diabetes, type 2 diabetes, and gestational diabetes [1]. T2DM is the most prevalent form, primarily due to insufficient insulin secretion or insulin resistance, which reduces tissue and organ sensitivity to insulin. It accounts for over 90% of all diabetes cases [2], with the number of patients continuously rising, which severely impacts public health and quality of life. While commonly used clinical drugs such as metformin, insulin (Ins), and glimepiride demonstrate significant hypoglycemic effects [3,4,5], their long-term use may induce adverse reactions in patients. Consequently, researchers remain dedicated to developing cost-effective, low-toxicity medications suitable for sustained diabetes management.

Platycodin D (PD), a pentacyclic triterpene saponin isolated from Platycodon root [6], exhibits a diverse array of pharmacological activities, including anti-inflammatory, antioxidant, hepatoprotective, hypoglycemic, and hypolipidemic effects [7,8,9,10,11]. Research indicates that PD can mitigate the rapid rise in blood glucose induced by high-sugar stimulation. It exerts an exogenous insulin-like effect without altering endogenous insulin levels, and its efficacy is comparable to that of metformin. PD regulates high-fat diet-induced hepatic lipogenesis via the AMPK signaling pathway while simultaneously reducing serum triglyceride levels. Furthermore, previous studies have demonstrated that PD can protect pancreatic islet cells in vitro from hyperglycemia-induced damage and reverse islet cell apoptosis [12]. Lee H et al. also found that PD upregulates the anti-adipogenic factor KLF2 (Krueper-like factor 2), which in turn downregulates PPARγ2 expression, ultimately producing a lipid-lowering effect [13]. Therefore, it holds great potential for the treatment of diabetes. However, PD is characterized by poor water solubility, a short in vivo half-life, and low bioavailability, which limit its clinical application. Consequently, exploring methods to modify the administration route of PD to enhance clinical compliance and improve its bioavailability has emerged as a key research focus. It is therefore essential to identify a safe and side-effect-free approach to enhance the bioavailability of PD, thereby broadening its applicability in diabetes treatment.

In recent years, nanoscale drug delivery systems have demonstrated tremendous potential in treating diabetes. Nanocarriers can target drugs to specific regions, addressing the challenge of insufficient drug delivery to particular sites and enhancing therapeutic efficacy [14]. Studies have shown that microsphere formulations protect drugs from degradation in the harsh gastric environment, improve drug bioavailability, and enhance the efficacy of oral medications. Among various formulations, alginate microspheres have gained the widest application due to their excellent biocompatibility, low cost, and pH sensitivity [15,16,17]. Based on this analysis, we hypothesize that alginate gel microspheres serve as an optimal carrier for loading PD. This approach is expected to increase the bioavailability of PD, thereby more effectively mitigating diabetic damage.

This study employed electrospray technology to fabricate PD@MPs. The synthesized PD@MPs were characterized and validated for their antioxidant and anti-apoptotic properties. Subsequently, a T2DM mouse model was established to evaluate the therapeutic efficacy of PD@MP. Liver, kidney, and pancreatic tissues were examined to investigate the protective effects of PD@MPs against T2DM.

## 2. Results and Discussion

### 2.1. Characterization of PD@MPs

**Figure 1:**
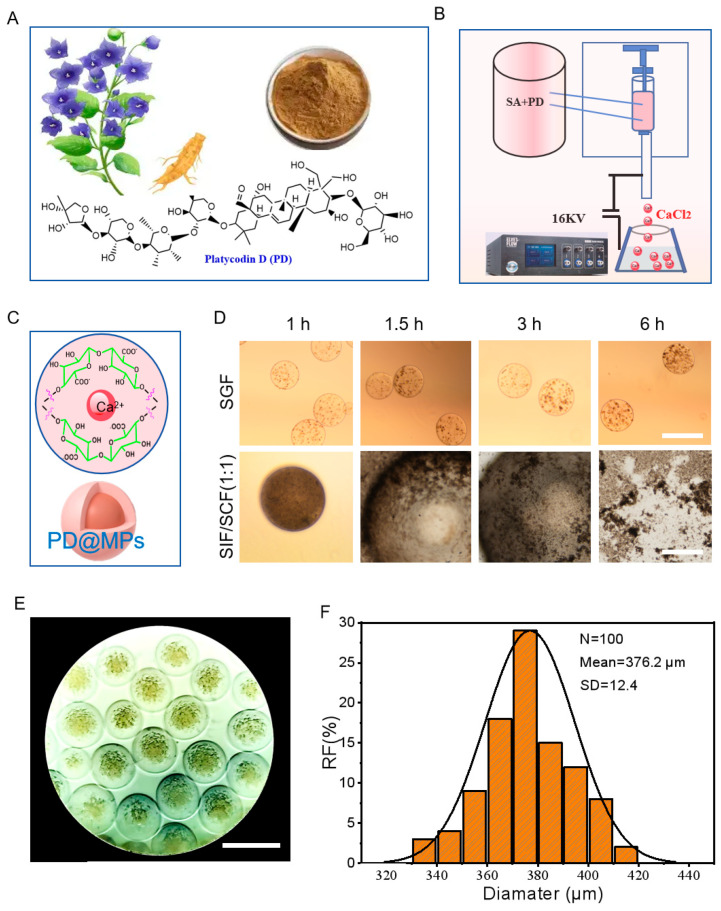
Structural characterization of PD@MPs. (**A**) The chemical structure formula of PD. (**B**) The schematic diagram of the preparation of PD@MPs. (**C**) The schematic diagram of the reaction between SA and Ca^2+^. (**D**) The stability test analysis of PD@MPs under gastrointestinal fluid, with a scale of 500 μm. (**E**) The microscopic observation image of the prepared PD@MPs, with a scale of 500 μm. (**F**) Particle size analysis of PD@MPs, *n* = 100, SD = 12.4.

Figure 1A shows the botanical morphology and chemical molecular formula of PD@MPs. (Figure 1B) depicts the preparation process of PD@MPs, which are produced via electrospinning. The mechanism of the reaction between Platycodin D and calcium chloride is shown in (Figure 1C). High sphericity is an essential requirement for drug carriers. Compared to irregular microparticles, microspheres with high sphericity exhibit more stable drug release, making them particularly suitable for treating conditions requiring long-term medication. Highly spherical microspheres facilitate precise drug dosage calculation, simplify administration, and exhibit superior flowability [18,19]. Thus, high sphericity, uniform particle size, and excellent dispersibility serve as key criteria for evaluating microsphere quality. (Figure 1E) schematically illustrates the microsphere formation process involving PD reacting with calcium chloride and SA. The PD@MPs prepared via electrospray technology exhibit high sphericity, uniform particle size, and excellent dispersibility. Additionally, microsphere stability testing (Figure 1D) indicates that PD@MPs remain stable in SGF for up to 6 h but degrade within 3 h in SIF. Even after 6 h, PD@MPs remained detectable in SGF. The intestine-targeted delivery capability of PD@MPs is governed by the pH-responsive ionization and conformational behavior of the SA matrix. In acidic gastric fluid, protonation of alginate carboxylate groups (-COO^−^) induces polymer chain collapse, forming a dense, impermeable hydrogel that protects encapsulated PD from degradation. Upon reaching the neutral/weakly alkaline intestinal environment, deprotonation of carboxylate moieties generates electrostatic repulsion, driving hydrogel swelling and sustained PD release. This pH-dependent behavior enables effective gastric protection and intestinal-specific targeted delivery of PD. We randomly selected the prepared PD@MPs microspheres and measured their particle size, obtaining an average value of 376.2 μm (Figure 1F).

### 2.2. PD Inhibits Cell Apoptosis, Oxidizes, and Enhances Cellular Activity

**Figure 2:**
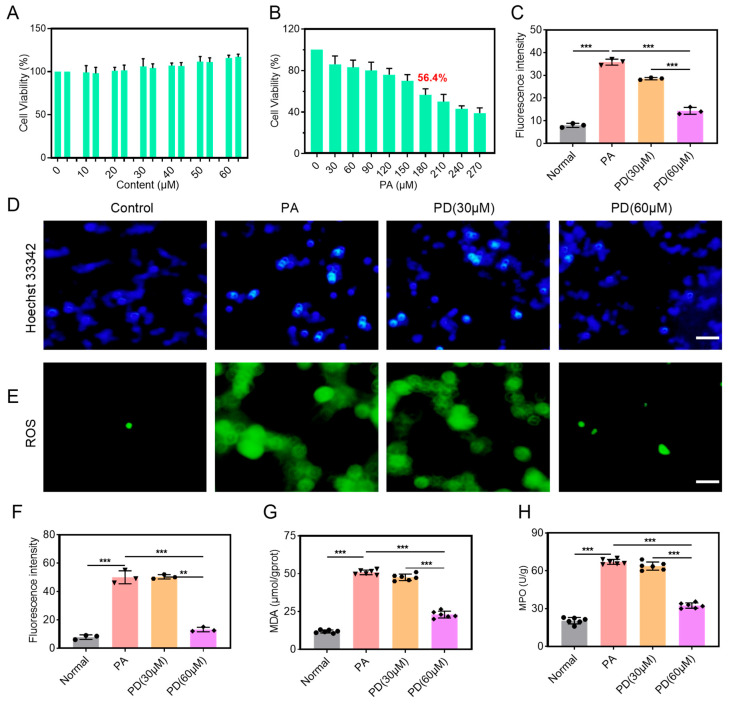
Results of in vitro anti-inflammatory and antioxidant activity studies. (**A**) Evaluation of the biological safety of PD. (**B**) Investigation of the dosage for PA modeling. (**C**) Quantitative analysis of the fluorescence intensity of Hoechst 33342. (**D**,**E**) Fluorescence staining analysis of ROS and Hoechst 33342. (**F**) Quantitative analysis of the fluorescence intensity of ROS. (**G**,**H**) Analysis of oxidative indicators MDA and MPO. The values represent the mean ± SD (*n* = 6). Statistical significance is indicated as * *p* < 0.05, ** *p* < 0.01, *** *p* < 0.001.

The occurrence of oxidative stress is a major cause of insulin resistance. Research suggests that insulin resistance is associated with the body’s physiological defense against excessive oxygen-free radicals [20]. Oxidative stress can interfere with insulin-receptor binding by activating stress-sensitive signaling pathways such as intracellular inflammatory pathways, protein kinases, and c-Jun N-terminal kinase (JNK). interfering with the binding of insulin to insulin receptors (IRS) and thereby weakening insulin’s normal physiological effects, leading to and exacerbating insulin resistance [21,22,23]. Therefore, diabetes is closely related to oxidative stress, and the levels of MPO and MDA can reflect indicators of the body’s antioxidant and oxidative status [24,25]. Here, we established an in vitro high-glucose cell inflammation model to evaluate the intervention effect of PD on the inflammation of L-02 cells. Firstly, when PD was applied to the cells, it was observed that no toxic side effects were shown within the dose range of 0–60 μM, and it had a certain promoting effect on cell proliferation (Figure 2A). Additionally, by using palmitic acid (PA) to induce cell inflammation, it was found that at a dose of 180 μM, the cell survival rate was reduced to 56.4%; therefore, this dose was selected for the subsequent experiments (Figure 2B). This study further explored the potential of PD to reverse cell apoptosis and oxidative stress induced by PA. Hoechst 33342 staining indicated that PD effectively inhibited the PA-induced cell inflammation and apoptosis (apoptotic cells showed bright and dense blue staining) phenomenon (Figure 2C,D). ROS staining revealed that PD could effectively reverse the occurrence of oxidative stress. Notably, the measurements of MDA and MPO in the cells showed that PD treatment significantly regulated the oxidative level, proving that PD has a significant preventive effect on oxidative damage induced by PA and demonstrating its strong antioxidant and anti-apoptotic properties (Figure 2G,H). In summary, these research results indicate that PD can alleviate oxidative stress-mediated cell damage caused by PA and exhibits a strong anti-apoptotic effect.

### 2.3. PD@MPs Effectively Alleviated STZ-Induced Hyperglycemia

**Figure 3:**
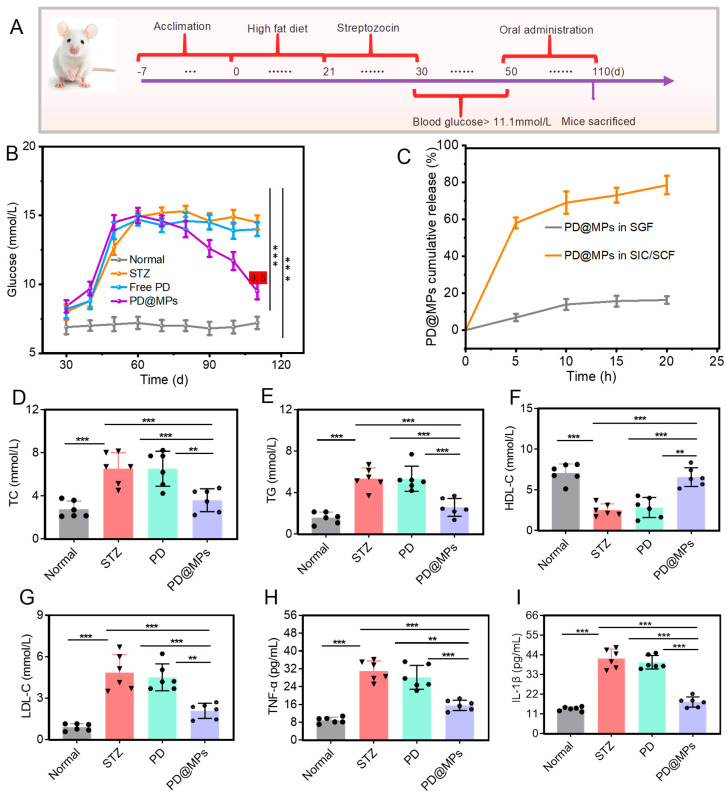
PD@MPS shows positive effects on diabetic mice. (**A**) Diagram of the feeding and experimental process of T2DM mice. (**B**) Blood glucose levels of mice were monitored after treatment with different drugs at different time intervals. (**C**) PD@MPs were released cumulatively in SGF and SIF/SCF (1:1). (**D**–**G**) Four blood lipids, including TC, TG, HDL-C, and LDL-C. (**H**,**I**) Inflammation indicators: TNF-α and IL-1β. The values represent the mean ± SD (n = 6). Statistical significance is indicated as ** *p* < 0.01, *** *p* < 0.001.

We established a diabetic model by inducing mice with a high-fat diet (HFD) combined with subcutaneous injections of STZ [26,27,28]. After a 7-day adaptation period, mice received a high-fat diet for 30 days (with a standard diet for the blank control group), receiving weekly STZ injections totaling three doses. After 10 days of stable blood glucose, fasting blood glucose values exceeding 11.1 mmol/L were selected as the modeling success criterion [29,30]. As shown in (Figure 3A), 50 days after administration of different formulations (Figure 3B), the results indicated that the free PD group and the STZ group had no hypoglycemic effect, while the PD@MPs group significantly improved abnormal blood sugar levels, reducing them to 9.5 mmol/L. At the same time, the in vitro drug release test showed that the cumulative release amount of PD@MPs in the intestine within 20 h exceeded 78.5%, while the release amount in the stomach was less than 20% (Figure 3C), confirming its pH-responsive release property and potential intestinal targeting property. Chronic hyperglycemia often results in abnormal blood lipid levels and an increase in inflammatory markers. This study examined the serum levels of total cholesterol (TC), triglycerides (TG), high-density lipoprotein cholesterol (HDL-C), and low-density lipoprotein cholesterol (LDL-C) in significant hypoglycemic and hypolipidemic effects of PD@MPs. As shown in (Figure 3D–G), compared with the normal control group, levels of TC, TG, and LDL-C were significantly elevated in the model group. However, following treatment with PD@MPs, levels of TC, TG, and LDL-C were significantly reduced in T2DM mice. PD@MPs can significantly alleviate lipid metabolism abnormalities in T2DM mice. To investigate the potential anti-inflammatory properties of PD@MPs in diabetic mice, the levels of tumor necrosis factor (TNF-α) and interleukin-1β (IL-1β) were measured using the ELISA assay. These data indicate that the inflammatory response was significantly attenuated following administration of PD@MPs (Figure 3H,I); compared with the normal control group, the levels of TNF-α and IL-1β were significantly elevated in the model group. However, following treatment with PD@MPs, TNF-α and IL-1β levels in T2DM mice were significantly reduced. PD@MPs can substantially alleviate inflammation in T2DM mice. This highlights its encouraging therapeutic potential.

### 2.4. PD@MPs Alleviated STZ-Induced Related Complications

**Figure 4:**
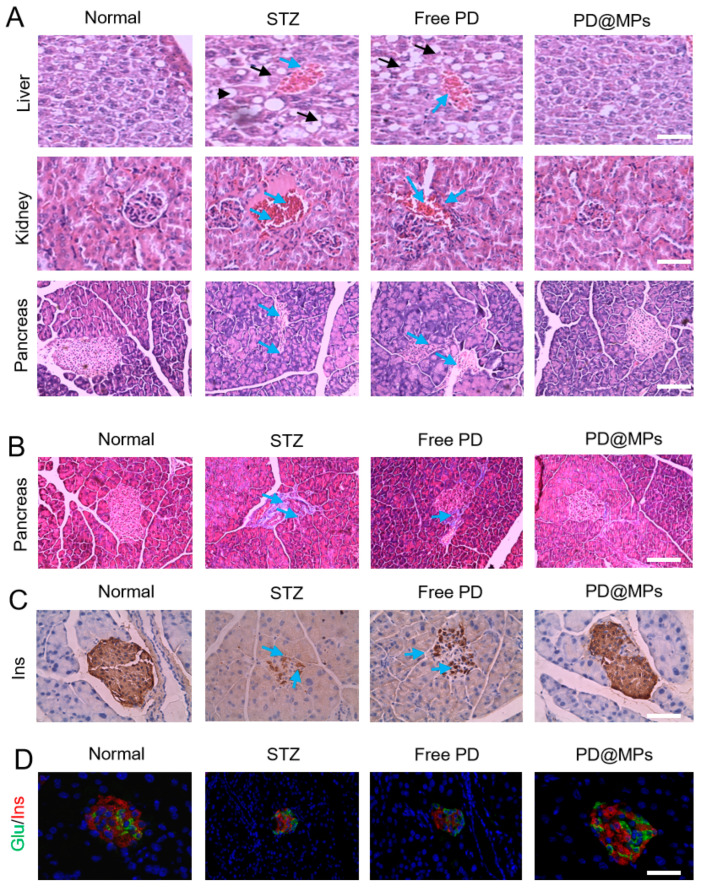
Histological section staining analysis. (**A**) H&E staining of liver, kidney, and pancreas tissues after treatment with different dosage forms. (**B**) The protective effects of PD@MPs on the pancreas were confirmed through Masson staining of tissue sections. (**C**) Proportion of glucagon in islets in immunohistochemical staining. (**D**) The effects of PD@MPs on islet morphology and inflammation were detected by immunofluorescence.

Diabetes can lead to various complications, including diabetic liver disease and diabetic nephropathy. This study aimed to investigate pathological changes in key organs, including the liver, kidneys, and pancreas. Histological examination of hepatic and renal tissues from mice, using H&E staining, revealed that in the blank control group, liver cells exhibited a radial distribution pattern with neat, compact, and clearly defined cell boundaries. The cytoplasm appeared plump and morphologically normal. In contrast, the T2DM model group exhibited diffuse vacuolar lipid accumulation in the liver, with the cytoplasm filled with small lipid droplets and accompanied by severe fatty degeneration. Following administration of PD, the lipid vacuoles and steatosis in the liver were significantly improved. After treatment with PD@MPs, liver morphology was indistinguishable from that of the normal group. This finding demonstrates that PD@MPs can effectively treat liver tissue damage induced by T2DM. H&E staining revealed that the kidney tissue of the control group exhibited a compact and intact structure, characterized by a normal mesangial cell matrix, normal glomerular morphology and proportions, well-filled glomeruli, absence of inflammatory cell infiltration, and no significant tissue damage. In contrast, the model group mice, subjected to STZ injury for four weeks without treatment, displayed irregular mesangial cells and glomerular morphology in kidney tissue, thickened glomerular capillary basement membranes, widened mesangial areas, atrophied glomerular capillaries, enlarged glomerular cavities, tubular vacuolar degeneration, glomerular atrophy, a loose tissue structure, and inflammatory cell infiltration. The PD group exhibited a reduction in inflammation compared to the model group. Mice in the PD@MPs group, after four weeks of treatment following STZ injury, demonstrated partial recovery of mesangial cells and glomerular morphology in renal tissue. The glomerular basement membrane was thinned, mesangial widening was not prominent, glomeruli were well-perfused, and inflammatory cell infiltration was markedly reduced, resembling the normal group. This demonstrates that PD@MPs can effectively treat renal tissue damage induced by T2DM (Figure 4A). These changes were assessed using H&E and Masson’s trichrome staining on pancreatic sections. The morphology in the PD@MPs group closely resembled that of the normal group, indicating that PD@MPs reduced collagen fiber proliferation (indicated by arrows pointing to blue areas) in response to STZ-induced pancreatic injury, suppressed hepatic and renal inflammatory infiltration, and preserved islet morphology. These findings provide compelling evidence for the potential of PD@MPs in alleviating diabetic complications. Immunohistochemical staining confirmed the role of PD@MPs in inhibiting apoptosis, promoting islet cell regeneration, and mitigating damage in STZ-induced diabetic mice. Glucagon plays a crucial role in maintaining normal glucose homeostasis and regulating metabolic abnormalities (Figure 4B,C), particularly in diabetes [31]. To evaluate the potential protective effects of different formulations on islet function, pancreatic sections were double-stained for insulin (red) and glucagon (green) (Figure 4D). Experiments revealed that compared to the STZ and free PD groups, PD@MPs-treated mice exhibited larger, more regular islet areas in pancreatic tissue, with reduced green fluorescence representing glucagon throughout the islets. This indicates minimal inflammatory infiltration and preserved islet morphology in PD@MPs-treated mice. In contrast, severe immune cell infiltration was observed in the STZ and free PD-treated groups. The number of secretory cells in islets from the PD@MPs and control groups was comparable, demonstrating that PD@MPs effectively protect islet function and may offer novel therapeutic insights for future islet treatments.

### 2.5. Effects on Protein Expression

**Figure 5:**
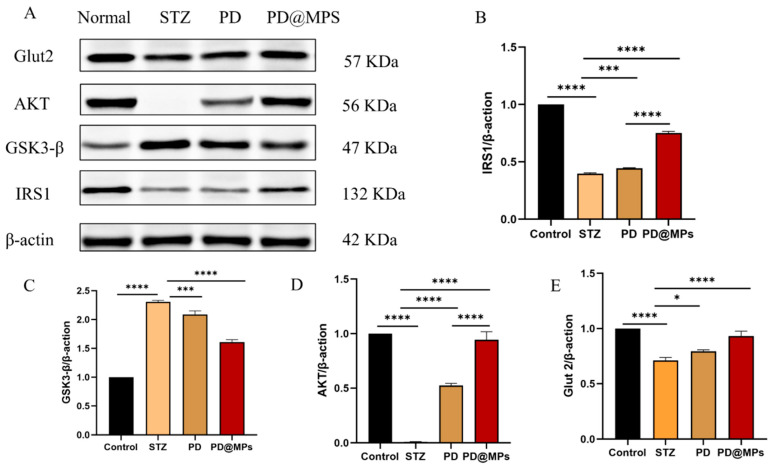
Effects of PD@MPs on related protein expression (**A**) Expressions of Glut2, GSK3-β, IRS1, and AKT in the liver were detected by Western blot. (**B**–**E**) protein expression levels of IRS1, Glut2, GSK3-β, AKT, and Glut2. Data are presented as the mean ± SD (n = 3), * *p* < 0.05, *** *p* < 0.001, **** *p* < 0.0001.

The liver plays a pivotal role in regulating blood glucose homeostasis by modulating glucose uptake, glycogen synthesis, and metabolic flux. Aberrant hepatic expression of AMP-activated GSK-3β is closely linked to insulin resistance, especially during the process of glycogen synthesis. Insulin resistance is a core driver in the pathogenesis of T2DM, with impaired tyrosine phosphorylation of IRS-1 widely recognized as a key biomarker for pancreatic insulin resistance in diabetic states. As illustrated in Figure 5A, Western blotting was performed to assess the protein expression levels of IRS-1, Glut2, GSK3-β, and AKT in liver tissue. (Figure 5B) demonstrates that IRS1 expression was significantly reduced in the STZ-induced model group relative to the normal control group. PD treatment partially restored IRS1 expression, whereas PD@MPs treatment exerted a more robust restorative effect, returning IRS1 levels to near-normal values that were significantly higher than those in the PD group. (Figure 5C) reveals that STZ induction markedly upregulated GSK3-β expression compared to the normal control, and both PD and PD@MPs treatments significantly blunted this overexpression, with PD@MPs displaying a stronger inhibitory efficacy. As presented in (Figure 5D), STZ treatment drastically suppressed AKT expression, and both PD and PD@MPs interventions effectively reversed this inhibition, restoring AKT levels to near-physiological levels. (Figure 5E) shows that Glut2 expression was significantly downregulated in the STZ group relative to the normal control. PD treatment partially restored Glut2 expression, while PD@MPs treatment elicited a more pronounced recovery, bringing Glut2 levels back to near-normal values that were significantly higher than those in the PD group. Collectively, these results demonstrate that PD@MPs treatment significantly reduces hepatic GSK3-β protein expression while upregulating IRS1, Glut2, and AKT expression in mouse liver tissue, indicating that PD@MPs therapy effectively alleviates insulin resistance and ameliorates hyperglycemia.

### 2.6. Biosafety Assessment

**Figure 6:**
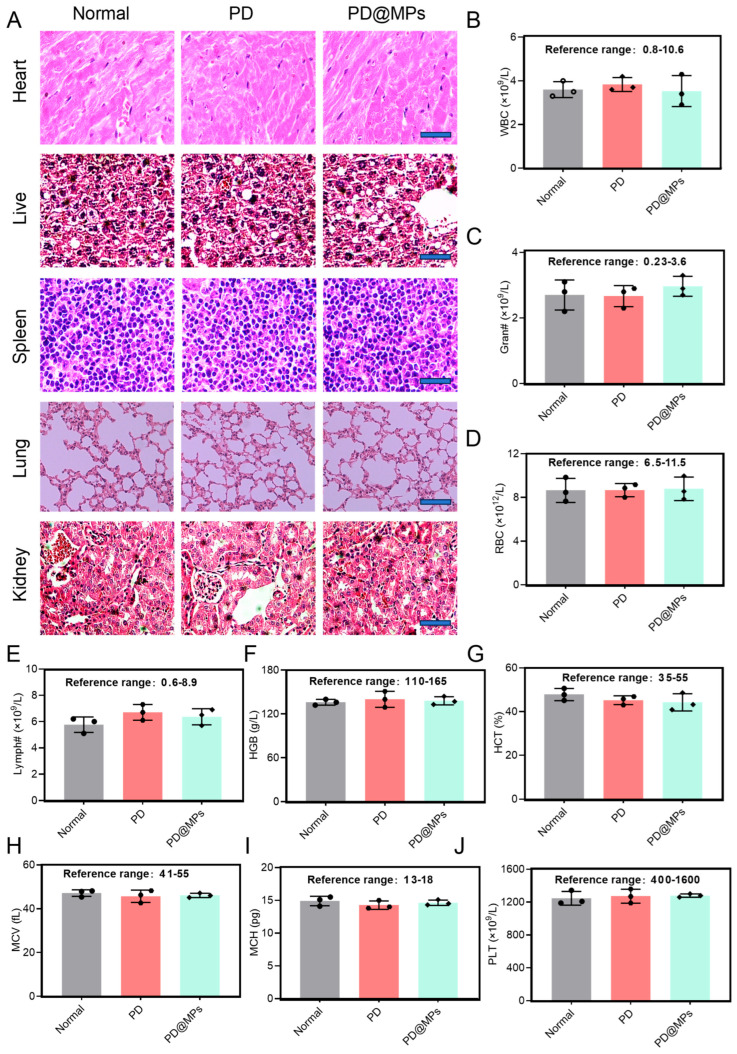
Evaluation of toxicology and tissue damage after oral administration of PD@MPs in mice. (**A**) H&E staining analysis of major organs treated with different samples. Scale bar: 50 μm. (**B**–**J**) WBC, white blood cells; Gran#, granulocyte; RBC, red blood cells; Lymp#, lymphocyte; HGB, hemoglobin; HCT, hematocrit; MCV, mean cell volume; MCH, mean corpuscular hemoglobin; PLT, blood platelet. The values represent the mean ± SD (n = 3).

Although our study confirmed the enhanced functionality of PD@MPs in T2DM, the safety of these preparations warrants careful consideration. Consequently, we conducted H&E analysis on the primary tissues associated with PD@MPs and evaluated basic serum biomarkers to assess the biosafety of each formulation. The H&E results indicated no significant alterations in the heart, liver, spleen, lung, and kidney tissues associated with these preparations (Figure 6A). Furthermore, serum physiological indices demonstrated that following treatment with each preparation, the white blood cell count (WBC), granulocytes (Gran#), red blood cell count (RBC), lymphocyte count (Lymph#), hemoglobin (HGB), hematocrit (HCT), mean red blood cell volume (MCV), mean red blood cell hemoglobin content (MCH), and platelet count (PLT) remained within the normal range and did not exhibit any abnormalities (Figure 6B–J).

## 3. Materials and Methods

### 3.1. Reagents and Kit

Platycodin D (Purity ≥ 99%), Streptozocin (STZ) (Shanghai Maclin Biochemical Technology Co., Ltd., Shanghai, China), microinjection pump (Guangjie Electronics Co., Ltd., Ningbo, China), high-voltage DC power supply (Dongwen High-Voltage Power Supply Co., Ltd., Tianjin, China), optical microscope (Leica Microsystems GmbH, Wetzlar, Germany), pH meter (Mettler Toledo Instruments Co., Ltd., Shanghai, China), GA-3 Sannuo blood glucose meter (Sannuo Biosensor Co., Ltd., Changsha, China), sodium alginate (SA) (Shanghai Aladdin Biochemical Technology Co., Ltd., Shanghai, China), anhydrous calcium chloride (Shanghai Maclin Biochemical Technology Co., Ltd.), Picric Acid-Acid Fuchsin compound stain, Aniline Blue stain, Digital Water Bath, Hematoxylin–Eosin stain, Malondialdehyde (MDA) assay kit, MPO assay kit (Shanghai Zhuocai Biotechnology Co., Ltd., Shanghai, China), Goat Anti-Rabbit IgG (H+L) HRP (Abclonal), β-actin Glut2 GSK3-β, IRS1 AKT (Abclonal) microplate reader (Meigu Molecular Instrument Co., Ltd., Shanghai, China), automatic washer (Shenzhen Huison Technology Development Co., Ltd., Shenzhen, China), water purifier (Sichuan Shui Siyuan Environmental Technology Co., Ltd., Chengdu, China), pipette (Dalong Xingchuang Laboratory Instrument Co., Ltd., Beijing, China), CO_2_ incubator (Thermo Fisher Scientific, Waltham, MA, USA), inverted fluorescence microscope (OLYMPUS, Tokyo, Japan), color prestained protein marker (10–180 kDa), kit for automatic glue making instrument (Servicebio, Wuhan, China), PVDF membrane (0.45 μm), (Servicebio), BCA protein concentration assay Kit (Biosharp, Beijing, China), SDS-PAGE protein loading buffer (5×) (Biosharp), phosphatase inhibitor 100× (Biosharp), PMSF (Biosharp), protease inhibitor 100× (Biosharp), cell lysis buffer for Western and IP (Beyotime, Shanghai, China), low temperature high speed tissue grinding instrument (Servicebio).

### 3.2. Preparation of PD@MPs Using Electrostatic Spray Technology

A total mass of 150 mg of PD was dispersed into 10 mL of a 1.5% (*w*/*v*) SA solution. The mixture was placed in a digital electronic water bath and stirred continuously at 50 °C until the solution became transparent. The resulting solution was transferred to a 5 mL syringe. The syringe was connected to a 26-gauge needle, which was attached to a voltage generator via an alligator clip, with 15 kV applied. Using a laboratory micro-syringe pump, the SA solution was introduced as droplets into a 2% (*w*/*v*) calcium chloride solution at a flow rate of 200 µL/min, with the needle positioned 9 cm above the receiving chamber. The SA solution was cross-linked with calcium ions to form gel microspheres, simultaneously encapsulating PD to yield PD@MPs.

### 3.3. Drug Loading Amount and Encapsulation Rate

During our production process, all the added PD could be effectively loaded into the microspheres. Therefore, the theoretical encapsulation rate is 100%. Regarding the drug loading amount, we conducted an analysis using high-performance liquid chromatography. We precisely weighed 2 g of Gas@MPs, thoroughly ground them, and then added 20 mL of methanol for dissolution. A total volume of 200 μL was taken for the high-performance liquid chromatography test. The conditions were: C18 chromatographic column, mobile phase of (acetonitrile: water = 25:75), flow rate of 1.0 mL/min, column temperature of 30 °C, injection volume of 10 μL, detection wavelength of 210 nm. Finally, the drug loading amount was calculated to be approximately 40.1%.

### 3.4. The Microscopic Morphology of PD@MPs Was Analyzed Using Transmission Electron Microscopy and Scanning Electron Microscopy

The prepared microsphere sample was placed in a clean Petri dish and positioned on the microscope stage. The microscope’s illumination, coarse focus knob, and fine focus knob were adjusted to achieve a clear field of view. Bright-field images were captured and saved. The optical microscope was switched to low magnification to observe the microspheres, and photographs were taken for storage.

### 3.5. In Vitro Cell Experiments Were Conducted to Preliminarily Evaluate the Antioxidant and Anti-Inflammatory Properties of PD

L-02 cells were seeded at a density of 1 × 10^5^ cells per well in 6-well plates and cultured for 24 h until they adhered. At this point, palmitic acid (200 μM) was added, and the cells were cultured for an additional 24 h. Subsequently, the cells were treated with PD@MPs (120 μM) and incubated for 24 h. To visualize oxidative stress levels, cells were stained with a ROS assay kit according to the manufacturer’s instructions and incubated at 37 °C for 30 min. Concurrently, cell apoptosis was assessed by Hoechst 33342 staining. After treatment, L-02 cells were stained with Hoechst 33342 (10 μg/mL) for 5 min, washed with PBS, and observed under a fluorescence microscope. Apoptotic nuclei were quantified using Image-Pro Plus 7.0 software.

### 3.6. Simulate the Release Performance of PD@MPs Using Artificial Gastrointestinal Fluid

An appropriate amount of PD@MPs was placed in simulated gastric fluid (SGF) for 1, 1.5, 3, and 6 h, and in simulated intestinal fluid/simulated colonic fluid (SIF/SCF) for 1, 1.5, 3, and 6 h, respectively. The particle size of PD@MPs was then measured to assess their stability. Additionally, microstructural changes were observed after 1, 1.5, 3, and 6 h via transmission electron microscopy (TEM).

### 3.7. The Digestion Process of PD@MPs Was Simulated In Vitro

The PD@MPs samples were placed in centrifuge tubes containing simulated gastric fluid (pH = 2.0) and simulated intestinal fluid (pH = 6.8) for 20 h. The entire process was conducted in a shaking incubator at 37 ± 1 °C at 50 rpm. Release solutions were extracted at predetermined intervals (0, 5, 10, 15, 20 h) and replenished with an equal volume of the corresponding release medium. Absorbance was measured at 250 nm using a UV-visible spectrophotometer. PD content was determined using an established standard curve, and a release curve was plotted.

### 3.8. Evaluation of the Regulatory Effect of PD@MPs on Abnormal Blood Sugar Through Experiments on Small Animals

Eight-week-old male Balb/c mice (body weight 26 ± 3 g) were purchased from Hangzhou Medical College, quality certificate number SYXK (Zhe) 2023–0015 (Hangzhou, China). After 7 days of acclimation feeding, mice were randomly assigned to a standard diet group and a high-fat diet group, with the standard diet group designated as the control group. Mice receiving a high-fat diet received intraperitoneal injections of STZ (50 mg/kg body weight) for ten days at intervals. Blood glucose levels exceeding 11.1 mmol/L were used as the criterion for successful modeling. Mice successfully modeled were divided into four treatment groups as follows: Normal group: saline solution; Model group: STZ injection solution (35 mg/kg); Free PD group (2 mg/kg); PD@MPs group (2 mg/kg). Both the positive control group and the treatment group received oral gavage once daily. Fasting blood glucose levels were measured weekly by collecting blood from the tail using a lancet.

### 3.9. Tissue Section Staining and Immunohistochemistry Analysis

On the final day of the experiment, mice were euthanized. The livers, kidneys, and pancreases from each group were removed and immersed in 4% paraformaldehyde. The remaining halves of the liver, kidney, and pancreas tissues were rapidly pre-chilled in liquid nitrogen and stored at −80 °C for subsequent use. The liver, kidney, and pancreas, immersed in 4% paraformaldehyde solution, were paraffin-embedded and sectioned into 4 μm thick slices. Fixed sections and processed sections were stained with H&E reagent according to the standard protocol in the manual. Finally, the specimens were observed under an optical microscope. Pancreatic tissue immersed in 4% paraformaldehyde solution was paraffin-embedded and sectioned into 4 μm thick slices. Fixed and processed sections were stained with Masson’s trichrome solution according to the manufacturer’s standard protocol. Finally, specimens were observed under a light microscope. Take paraffin-embedded pancreatic tissue sections and perform dewaxing following the same steps as for H&E staining. After rinsing in distilled water, the sections were incubated with a 3% hydrogen peroxide solution to block endogenous peroxidase activity at 37 °C for 30 min. Antigen retrieval was performed by heating the sections in citrate buffer (pH 6.0) in a microwave oven at medium-high power for 8 min. After cooling to room temperature, the sections were blocked with 5% BSA for 10 min. Subsequently, the sections were incubated overnight at 4 °C with primary antibodies against PCK1, NF-κB, and Caspase-3, each at a dilution of 1:300. After washing, the sections were incubated with polymer-conjugated HRP-labeled goat anti-rabbit IgG secondary antibody at 37 °C for 30 min. After washing three times with PBS, the sections were incubated with DyLight-conjugated secondary antibody (1:200) in the dark for 30 min. After three washes with PBS, the slides were mounted with anti-fade mounting medium, and immunostaining was observed under a fluorescence microscope. Perform fluorescence intensity analysis. The expression levels were quantified using Image-Pro Plus 7.0 software.

### 3.10. Analysis of the Mechanism of Action of PD@MPs on T2DM

The target tissue was removed and washed with pre-chilled 1× PBS to remove residual blood. The tissue was minced into small pieces on ice, and the tissue fragments were placed in a grinding tube containing 2–3 grinding beads. The protein concentration was determined using the BCA assay. The samples were loaded into the electrophoresis wells. Electrophoresis was performed at 80 V for the stacking gel and 120 V for the separating gel and continued until the bromophenol blue dye reached the bottom of the gel. Following electrophoretic separation, proteins were transferred to a PVDF membrane, which was blocked with 5% non-fat milk for 1 h at room temperature. The membrane was then incubated with the prepared primary antibody overnight at 4 °C. After washing, the membrane was incubated with the secondary antibody solution for 1 h at room temperature. The secondary antibody solution was discarded, and the membrane was washed again. Finally, the developing solution was applied, and images were captured using the Qinxiang Chemiluminescence Imaging System. Band grayscale values were analyzed using Image-Pro Plus 7.0 software, and the relative expression levels of the target protein were calculated.

### 3.11. Statistical Analysis

Data are presented as the mean ± standard deviation (mean ± SD). One-way ANOVA followed by Bonferroni’s post-hoc test was employed to assess statistical significance. The significance levels were denoted as follows: ns’ indicates *p* > 0.05 (no significance, ns), * *p* < 0.05 indicates significance, ** *p* < 0.01 indicates high significance, and *** *p* < 0.001 indicates very high significance. **** *p* < 0.0001.

## 4. Conclusions

In this study, we successfully fabricated pH-responsive, intestine-targeted PD@MPs via electrospray technology using SA as a biocompatible carrier. PD@MPs exhibited high sphericity, uniform particle size, and efficient PD loading, with a pH-dependent release profile that protects PD from gastric degradation and enables sustained intestinal release. In vitro experiments confirmed that PD@MPs retained PD’s antioxidant and anti-apoptotic activities, attenuating PA-induced oxidative damage in L-02 cells. In vivo studies demonstrated that PD@MPs exerted superior hypoglycemic effects in HFD/STZ-induced T2DM mice, ameliorated inflammatory injury in liver and kidney tissues, and preserved pancreatic islet structure and function by inhibiting gluconeogenesis, inflammation, and apoptosis. Collectively, this work provides a novel oral delivery strategy to enhance PD’s bioavailability and therapeutic efficacy against T2DM, offering a promising platform for the clinical translation of natural anti-diabetic agents. Nevertheless, the present study has several limitations. No assessment was performed regarding interspecies variations in gastrointestinal pH, gut microbiota, and drug absorption, which may limit translational relevance. Moreover, the pharmacokinetic and biodistribution profiles of PD@MPs have not been comprehensively defined, and the long-term toxicity and immunogenic potential of the alginate carrier remain unclear. While pH-responsive release was achieved, the formulation’s in vivo targeting efficiency toward the intestinal epithelium and pancreatic islets requires direct validation. Future work will focus on PD@MPs formulation optimization (e.g., alginate modification with targeting ligands) and evaluation of long-term safety and pharmacokinetics in large animal models. Further studies will also investigate PD@MPs-gut microbiota interactions, assess effects on intestinal barrier function and systemic immunity, and develop scalable production strategies to advance clinical translation.

## Data Availability

The data that support the findings of this study are available from the corresponding authors upon reasonable request.
